# Return-to-Work Prevalence Among COVID-19 Patients After Receiving Intensive Care: A Systematic Review and Meta-Analysis

**DOI:** 10.7759/cureus.46315

**Published:** 2023-10-01

**Authors:** Takeshi Unoki, Hideaki Sakuramoto, Akira Ouchi, Noriko Inagaki, Mio Kitayama, Yusuke Kawai, Tomomi Furumaya, Yoko Tsukada

**Affiliations:** 1 Department of Acute and Critical Care Nursing, School of Nursing, Sapporo City University, Sapporo, JPN; 2 Department of Critical Care and Disaster Nursing, Japanese Red Cross Kyushu International College of Nursing, Munakata, JPN; 3 Department of Adult Health Nursing, College of Nursing, Ibaraki Christian University, Hitachi, JPN; 4 Faculty of Nursing, Setsunan University, Hirakata, JPN; 5 Department of Nursing, Kanazawa Medical University Hospital, Uchinada, JPN; 6 Department of Nursing, Fujita Health University Hospital, Toyoake, JPN; 7 Department of Nursing, Saitama Red Cross Hospital, Saitama, JPN; 8 Department of Nursing, Tokyo Medical and Dental University Hospital, Tokyo, JPN

**Keywords:** jobless, critical illness, covid-19, return-to-work, intensive care

## Abstract

Returning to work can be a serious issue for patients who have undergone intensive care. Previous studies have reported a relatively low return-to-work prevalence among such patients. Some patients with coronavirus disease 2019 (COVID-19) experience severe pneumonia and require intensive care, including mechanical ventilation. However, little is known about the return-to-work prevalence among such patients. Therefore, we conducted a systematic review and meta-analysis of the literature describing the return-to-work prevalence among COVID-19 patients who received intensive care. The eligibility criteria were determined based on the medical condition, context, and population framework of each study, as follows: (1) full-text observational studies, (2) context: COVID-19 patients admitted to ICU, (3) condition: return-to-work prevalence after ICU discharge, and (4) population: critically ill patients who are 18 years and older. Eligible studies included randomized controlled trials (RCTs) and observational studies. Review articles, case reports, letters to the editor, and comments without data involving return-to-work prevalence were excluded.

We searched the Medical Literature Analysis and Retrieval System Online (MEDLINE, via PubMed), the Cumulated Index to Nursing and Allied Health Literature (CINAHL, via EBSCOhost), and the International Clinical Trials Registry Platform (ICTRP) databases from their inception till July 26, 2022, and updated the search on June 14, 2023. Specifically, we collected studies reporting data on the return-to-work prevalence among COVID-19 patients after receiving intensive care. Data extraction and quality assessment were performed using the Joanna Briggs Institute (JBI) Critical Appraisal Checklist for Prevalence Studies. Pre-developed standard forms were used for data collection, and pooled prevalence for return-to-work was calculated. Out of the 2221 available records, 42 full texts were reviewed, 20 of which were included in the qualitative synthesis. The number of return-to-work cases reported at 0-3 months, 4-6 months, and 7-12 months were three, 11, and nine, respectively. At 0-3 months, the pooled prevalence was 0.49 (three trials; n = 73; 95% CI: 0.15-0.84; I^2^ = 82%). At 4-6 months, the pooled prevalence was 0.57 (11 trials; n = 900; 95% CI: 0.40-0.73; I^2^ = 92%). Finally, at 7-12 months, the pooled prevalence was 0.64 (nine trials; n = 281; 95% CI: 0.50-0.77; I^2^ = 80%). However, the overall quality of the included studies was low. Based on the results, approximately one-third of COVID-19 patients did not return to work 12 months after receiving intensive care. Given the quality and limitations of the studies, a more detailed and extensive cohort study is required; also, concerned authorities should implement adequate measures in terms of providing integrated job support for this patient population.

## Introduction and background

Post-intensive care syndrome (PICS) refers to the condition that occurs after and during intensive care, and its symptoms include physical dysfunction, mental health issues, and cognitive decline [1]. PICS can significantly impact the quality of life and return-to-work status of patients [2,3]. A previous meta-analysis has shown that the return-to-work prevalence of ICU patients was 60% a year from discharge [4]. Returning to work is associated with matters related to income, impaired mental health, and health-related quality of life [5,6].

Some patients with coronavirus disease 2019 (COVID-19) suffer from severe pneumonia and require intensive care, such as mechanical ventilation and extracorporeal membrane oxygenation (ECMO) [7]. Some of those patients experience PICS, similar to critically ill patients without COVID-19 [8]. Additionally, one study has reported that more than half of patients with COVID-19 who received mobility ECMO had pain/discomfort, mobility problems, and difficulty performing their routine daily activities [9]. We hypothesized that PICS would be associated with return-to-work in patients who received intensive care for COVID-19.

Furthermore, Long COVID, which entails fatigue and muscular weakness, is characterized by persistent symptoms following COVID-19 infection [10]. We considered that these symptoms may be associated with the return-to-work status (being employed and actively working) of patients who have recovered from (or are recovering from) COVID-19. Thus, for the patients who received intensive care, both PICS and Long COVID may decrease return-to-work prevalence. One systematic review concerning return-to-work in COVID-19 patients has been published. However, it did not undertake a meta-analysis [11], and nor did it focus on the patients who received intensive care [11]. Thus, there is a lack of understanding with regard to the long-term return-to-work prevalence following intensive care for COVID-19.

In light of this, we conducted a systematic review and meta-analysis to assess the return-to-work prevalence among adult patients who received intensive care for COVID-19.

## Review

Methods

This systematic review and meta-analysis was conducted by adhering to the guidelines of the Preferred Reporting Items for Systematic Reviews and Meta-analyses (PRISMA) statement [12]. The protocol for the systematic review was registered with the International Prospective Register of Systematic Reviews (PROSPERO; registration number: CRD42022346222).

Eligibility Criteria

The eligibility criteria were defined based on the condition, context, and population framework as follows: (1) full-text observational study, (2) context: patients with COVID-19 admitted to the ICU, (3) condition: return-to-work after ICU discharge, and (4) population: critically ill patients who are 18 years and older. Eligible studies included randomized controlled trials (RCTs) and observational studies. Review articles, case reports, letters to the editor, and comments without data involving return-to-work prevalence were excluded. If the data available to estimate the return-to-work prevalence was deemed insufficient in a particular article, the corresponding authors of those articles were contacted. Furthermore, we excluded studies wherein the evaluation period for return-to-work prevalence was unknown or was too broadly defined.

Information Sources and Search Strategy

We searched the Medical Literature Analysis and Retrieval System Online (MEDLINE, via PubMed), the Cumulated Index to Nursing and Allied Health Literature (CINAHL, via EBSCOhost), and the International Clinical Trials Registry Platform (ICTRP) databases from inception to July 26, 2022, and updated the search on June 14, 2023. The following search terms were used on all the databases: “critical care,” “intensive care unit,” “critical illness,” “coronavirus disease 2019,” “return-to-work,” “unemployment,” “sick leave,” “working,” and “jobless.” These search terms were derived from MeSH or Entry terminology. Manual searches were performed on Google Scholar. No language restrictions were imposed.

Selection Process

Of the eight reviewers, two independently reviewed the study titles and abstracts to identify potentially relevant studies. Subsequently, two reviewers independently assessed the studies’ eligibility based on a full-text review. Disagreements between the reviewers were resolved through a consensus discussion. If the disagreement remained unresolved, it was arbitrated by a third reviewer.

Data Collection Process

We extracted data on the authors’ names, year of publication, study design, country, sample size, the participants’ age range, and the severity of illness. Outcome data were categorized according to the return-to-work time frame after receiving intensive care. The categories were 0-3 months, 4-6 months, 7-12 months, and 13-24 months after ICU or hospital admission and discharge. Data were documented using a Microsoft Excel spreadsheet. Two reviewers independently extracted the data from individual studies in a dichotomous manner to pool the results. The results were pooled according to the category. 

Outcome

The main outcome measure of this study was the return-to-work prevalence among COVID-19 patients at various pre-defined time points after ICU discharge. For this study, "return to work" was defined as being employed before ICU admission and after ICU discharge. Because we speculated that some studies may not have provided detailed employment status, we assumed that if the patient was employed, it did at least mention if the workplace or work hours changed. We defined the status “not returning to work” as “being employed but genuinely not working”; individuals with such a status included those on long-term sick leave and those receiving employment benefits.

Assessment of Study Quality

The Joanna Briggs Institute (JBI) Critical Appraisal Checklist for Studies Reporting Prevalence Data was used to assess the methodological quality of the included studies [13]. This checklist contains nine questions. The questions are divided into three areas: participants (questions 1, 2, 4, and 9), outcome measures (6 and 7), and statistics (3, 5, and 8). In all three areas, a study was rated as high quality if it had an appropriate methodology. The risk of bias was independently evaluated by two reviewers. Disagreements among reviewers were resolved through consensus discussion; if they could not be resolved, they were arbitrated by a third reviewer.

Statistical Analysis

We pooled the prevalence estimates from the included studies by using a random-effects meta-analysis model. Meta-analysis was performed based on defined periods (e.g., 4-6 months, 7-12 months). Heterogeneity across studies was assessed using the I^2^ statistic.

Data from the longest follow-up period available for each study was used for the prevalence estimates. A subgroup analysis assessed whether the population of patients receiving ventilatory therapy only affected the estimates at the time of the return-to-work assessment. We used a sensitivity analysis to assess the robustness of the results by removing only the population that received ECMO. Additionally, a post-hoc analysis was conducted to clarify the heterogeneity of the primary analysis.

Certain policies are implemented by the governments or concerned authorities to compensate for disability and improve employment opportunities, making it a desirable choice for patients. These policies are implemented with the support of both workers and employers [14]. Many countries are shifting from compensation-driven support to a more integrated approach. We conducted a post-hoc subgroup analysis, defining countries with values above the median of the integration index for each Organization of Economic Cooperation and Development (OECD) country as having highly integrated support policies and countries below the median as having low integrated support policies [14]. In this analysis, we excluded articles from non-OECD countries [14].

The results are presented as forest plots with 95% confidence intervals (95% CI). The analyses were conducted using the R statistical software version 3.4.4, package “meta” version.

Results

Study Selection

**Figure 1:**
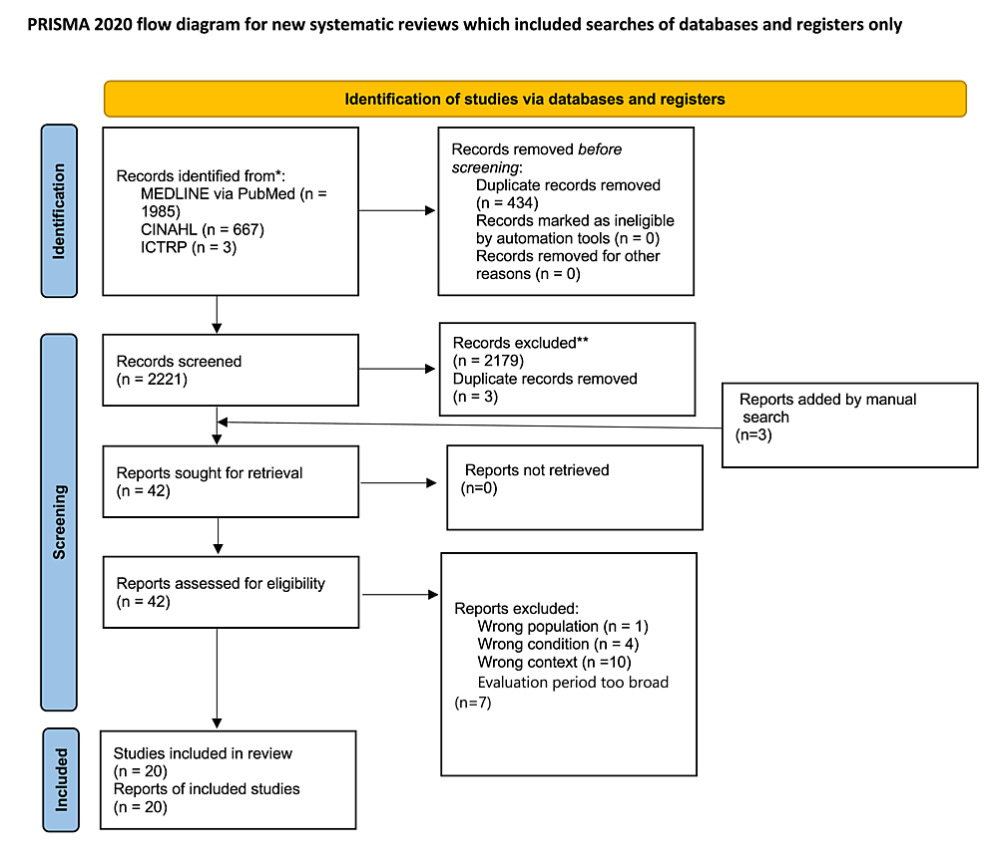
PRISMA flow chart depicting the selection of studies PRISMA: Preferred Reporting Items for Systematic Reviews and Meta-Analyses

The PRISMA flow diagram is shown in Figure 1. Our search initially yielded 2221 articles and abstracts. A total of 42 studies were assessed; 20 studies involving 1254 patients were included in the quantitative synthesis and 19 studies were used for the meta-analysis. The number of studies (i.e., number of patients) reporting an instance of return-to-work at 0-3 months, 4-6 months, and 7-12 months were three [15-17], eleven [18-27], and eight [17,25,27-32], respectively. Studies in which outcome assessments were conducted over an extended time frame were excluded.

Study Characteristics

All the included studies were published from 2021 to 2023 (Table 1). Two studies [23,32] were conducted in the United States, 16 in Europe [15,17,18,20-22,24-31,33,34], one in Brazil [16], and one in Australia [19]. Four studies [16,25,26,33] assessed return-to-work in patients receiving ECMO.

**Table 1:** Characteristics of the included studies IQR: interquartile range; SD: standard deviation; APACHE II: Acute Physiology and Chronic Health Evaluation II; APACHE IV: Acute Physiology and Chronic Health Evaluation IV; ICU: intensive care unit; SAPS: Simplified Acute Physiology Score; SOFA: Sequential Organ Failure Assessment

Author	Year	Country/region	Design	Study period	Population	Males, n (%)	Age, years, median (IQR) or mean ±SD	Severity of illness based on different scores, median (IQR) or mean ±SD	Number in employment prior to ICU admission	Males, n (%)	Age, years, median (IQR)	Evaluation period	Comments
Carenzo et al. [18]	2021	Italy	Prospective observational study	1 March 2020 to 30 June 2020	71 ICU patients	40 (89)	57 (51-62)	APACHE II: 6 (5-10)	45	38 (84.4)	57 (51-61)	6 months	
Demoule et al. [28]	2022	France	Prospective observational study	April 2000 to June 2020	94 ICU patients	67 (71)	63 (49-70)	SAPS 2: 33 (26-39)	44	-	-	12 months	12 patients were employed part-time
Fontes et al. [15]	2022	Portugal	Retrospective study	October 2020 to February 2021	99 ICU patients	63 (63.6%)	63 ±12	SAPS 2: 35 ±14	38	-	-	1 month	
Gilmartin et al. [22]	2022	Ireland	Prospective cohort study	-	22 ICU patients	15 (68)	52.4 ±15	-	14	-	-	6 months	
Schandl A et al. [20]	2021	Sweden	Prospective observational study	25 March to 13 August 2020	113 ICU patients	86 (76%)	-	-	46		<65	5 months	
van Veenendaal et al. [21]	2021	Netherlands	Prospective cohort study	19 March to 30 September 2020	60 ICU patients	41 (68%)	62.5 (55.3-68.0)	APACHE Ⅳ: 55.0 (45.0-65.3)	30	-	-	6 months	Four patients had reduced work rates, three patients had occupation change
Vitoria Pérez et al. [24]	2022	Spain	Prospective observational study	12 March to 31 December 2020	73 ICU patients	46 (60.5%)	61.8 ±8.8	APACHE Ⅱ: 19.7 ±7.1	30			4-5 months	
Zangrillo et al. [29]	2022	Italia	Prospective observational study	25 February to 27 April 2020	56 ICU patients	50 (89%)	56 ±11.9	-	38	-	-	12 months	
Hodgson et al. [19]	2021	Australia	Multicenter, prospective cohort study	6 March to 4 October 2020	160 ICU patients	97 (60.6%)	62 (55-71)	APACHE Ⅱ: 15 (11-19)	114			6 months	
Neville et al. [23]	2022	United States	Prospective cohort study	11 March to 31 December 2020	205 ICU patients	118 (58%)	59.6 (48.2-70.3)	SOFA: 3.0 (0.0-7.0)	68	-	-	6 months	40/68 patients returned to work after 6 months; 32 fully returned to work
Guenther et al. [26]	2023	Germany	Retrospective cohort study with prospective follow-up	April 2020 to September 2021	60 ECMO patients	46 (76.7%)	60.5 (51.0-65.0)	-	-	-	-	6 months	1/9 patients returned to work after 6 months
Lorusso et al. [33]	2022	Europe	Prospective observational study	1 March 2020 to 13 September 2020	1215 ECMO patients	942 (78%)	53 (46-60)	-	-	-	-	6 months	102/431 patients returned to work; 57/428 patients were employed in part-time work
Galas et al. [16]	2023	Brazil	Retrospective cohort study	April 2020 to August 2021	85 ECMO patients	72 (84.7%)	59 ±13	SAPS 3: 54 ±12	-	-	-	1–3 months	5/11 patients returned to work at 1 month; 10/11 patients returned to work at 3 months
Wiertz et al. [30]	2022	Netherlands	Prospective cohort study	2 April 2020 to 30 June 2020	103 ICU patients	52 (77.6%)	62 (57-68)	-	36	-	-	12 months	18/36 patients returned to work at 12 months
Chommeloux et al. [25]	2022	France	Prospective observational study	1 March 2020 to 15 June 2020	62 ECMO patients	45 (72%)	47 (40-55)	SAPS 2: 45 (32-54)	-	-	-	6 months, 12 months	13/62 patients returned to work at 6 months; 19/50 patients returned to work at 12 months
Onrust et al. [31]	2023	Netherlands	Prospective cohort study	19 March 2020 to 30 September 2020	56 ICU patients	38 (68%)	62 (55-68)	APACHE 4: 55.0 (45.0-66.0)	36	-	-	12 months	29/36 patients returned to work at 12 months
Larsson et al. [27]	2023	Sweden	Prospective cohort study	13 March 2020 to 14 July 2020	46 ICU patients	34(74%)	59 (53-69)	SAPS 3: 52 (46-55)	32	-	-	4 months, 12 months	20/32 patients returned to work at 3-6 months; 22/32 patients returned to work at 12 months
Heraud et al. [32]	2023	United States	Prospective observational study	January to September 2020	44 ICU patients	20 (45.5%)	56.5 (47.3-63.0)	APACHE: 14.5 (10.5-20.8)	-	-	-	<1 year post-discharge, >1 year post-discharge	
Herrmann et al. [17]	2023	Germany	Prospective longitudinal study	March and December 2020	45 ICU patients	29 (64%)	61 (52-68)	-	-	-	-	3 months, 12 months	10/27 patients returned to work at 3 months; 11/30 patients returned to work at 12 months
Wahlgren et al. [34]	2023	Sweden	Prospective cohort study	1 March 2020 to 31 May 2020	47 ICU patients	38 (81%)	64 ±11	-	24	-	-	2 years	13/22 patients returned to work at 2 years

Evidence Quality

The risk of bias in the included studies is shown in Table 2. All studies had a high risk of bias in the participant and statistics domains. Furthermore, none of the studies were of high quality and none of them had a sufficient sample size or provided a detailed description of characteristics. We determined that all the studies had a low risk of bias in the outcome domain.

**Table 2:** Risk of bias in the included studies ^*^The questions addressed were as follows: 1. Was the sample frame appropriate to address the target population? 2. Were study participants sampled appropriately? 3. Was the sample size adequate? 4. Were the study participants and the setting described in detail? 5. Was the data analysis conducted with sufficient coverage of the identified sample? 6. Were valid methods used for the identification of the condition? 7. Was the condition measured in a standard, reliable way for all participants? 8. Was there an appropriate statistical analysis? 9. Are all important confounding factors/subgroups/differences identified and accounted for? 10. Were subpopulations identified using objective criteria?

Author	Year	Country/region	1^*^	2^*^	3^*^	4^*^	5^*^	6^*^	7^*^	8^*^	9^*^	10^*^
Carenzo et al. [18]	2021	Italy	Y	Y	N	N	Y	Y	Y	N	Y	NA
Hodgson et al. [19]	2021	Australia	Y	Y	N	Y	N	Y	Y	Y	N	NA
Schandl et al. [20]	2021	Sweden	Y	Y	N	Y	N	Y	Y	N	Y	N
van Veenendaal et al. [21]	2021	Netherlands	Y	Y	N	Y	N	Y	Y	N	N	NA
Demoule et al. [28]	2022	France	N	N	N	Y	N	Y	Y	N	N	NA
Fontes et al. [15]	2022	Portugal	Y	Y	N	Y	N	Y	Y	N	N	NA
Gilmartin et al. [22]	2022	Ireland	Y	Y	N	Y	Y	Y	Y	N	N	NA
Larsson et al. [27]	2022	Sweden	Y	Y	N	Y	Y	Y	Y	N	N	NA
Vitoria Pérez et al. [24]	2022	Spain	Y	N	N	Y	N	Y	Y	N	N	NA
Zangrillo et al. [29]	2022	Italia	Y	Y	N	Y	N	Y	Y	Y	N	NA
Wiertz et al. [30]	2022	Netherlands	Y	Y	N	Y	N	Y	Y	N	N	NA
Chommeloux et al. [25]	2022	France	Y	Y	N	Y	N	Y	Y	N	N	NA
Lorusso et al. [33]	2022	Europe	Y	Y	Y	Y	N	Y	Y	N	N	NA
Neville et al. [23]	2022	United States	Y	Y	N	Y	N	Y	Y	N	N	NA
Galas et al. [16]	2023	Brazil	Y	Y	N	Y	N	Y	Y	N	N	NA
Guenther et al. [26]	2023	Germany	Y	Y	N	Y	N	Y	Y	N	N	NA
Onrust et al. [31]	2023	Netherlands	Y	Y	N	Y	N	Y	Y	N	N	NA
Heraud et al. [32]	2023	United States	Y	Y	N	Y	N	Y	Y	N	N	NA
Herrmann et al. [17]	2023	Germany	Y	Y	N	Y	N	Y	Y	N	N	NA
Wahlgren et al. [34]	2023	Sweden	Y	Y	N	Y	N	Y	Y	N	N	NA

Results of Syntheses

**Figure 2:**
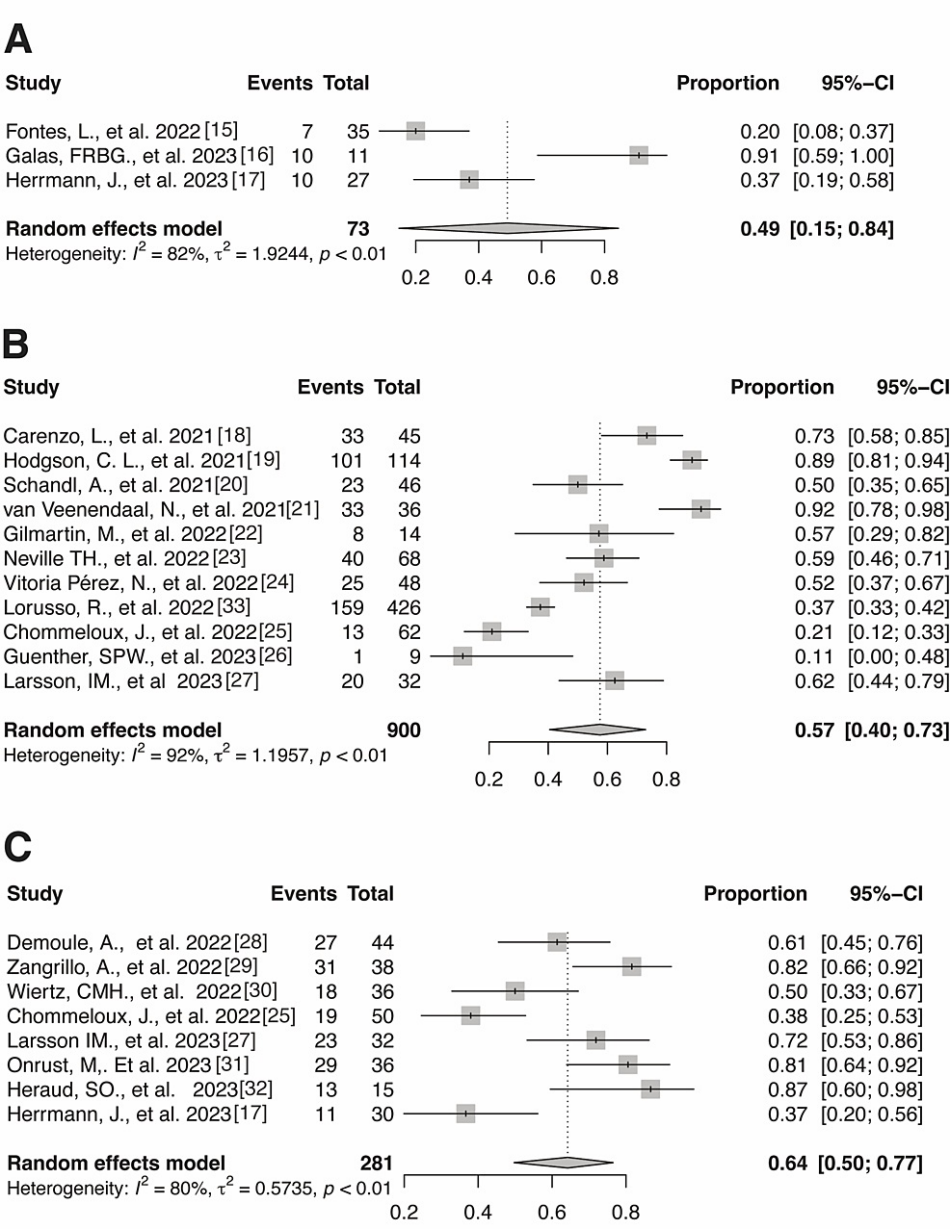
Forest plot of return-to-work prevalence after 0–3 months (A), 4–6 months (B), and 7–12 months (C)

The number of instances of return-to-work reported at 0-3 months, 4-6 months, and 7-12 months were four, seven, and three, respectively. At 0-3 months, the pooled prevalence was 0.49 (three trials; n = 73; 95% CI: 0.25-0.84; I^2^ = 82%) (Figure 2A). At 4-6 months, the pooled prevalence was 0.57 (11 trials; n = 900; 95% CI: 0.40-0.73; I^2^ = 92%) (Figure 2B). At 7-12 months, the pooled prevalence was 0.64 (eight trials; n = 281; 95% CI: 0.50-0.77; I^2^ = 80%) (Figure 2C). Notably, no meta-analysis was performed because only one study [34] had a follow-up after 13 months.

Subgroup Analysis and Sensitivity Analysis

**Figure 3:**
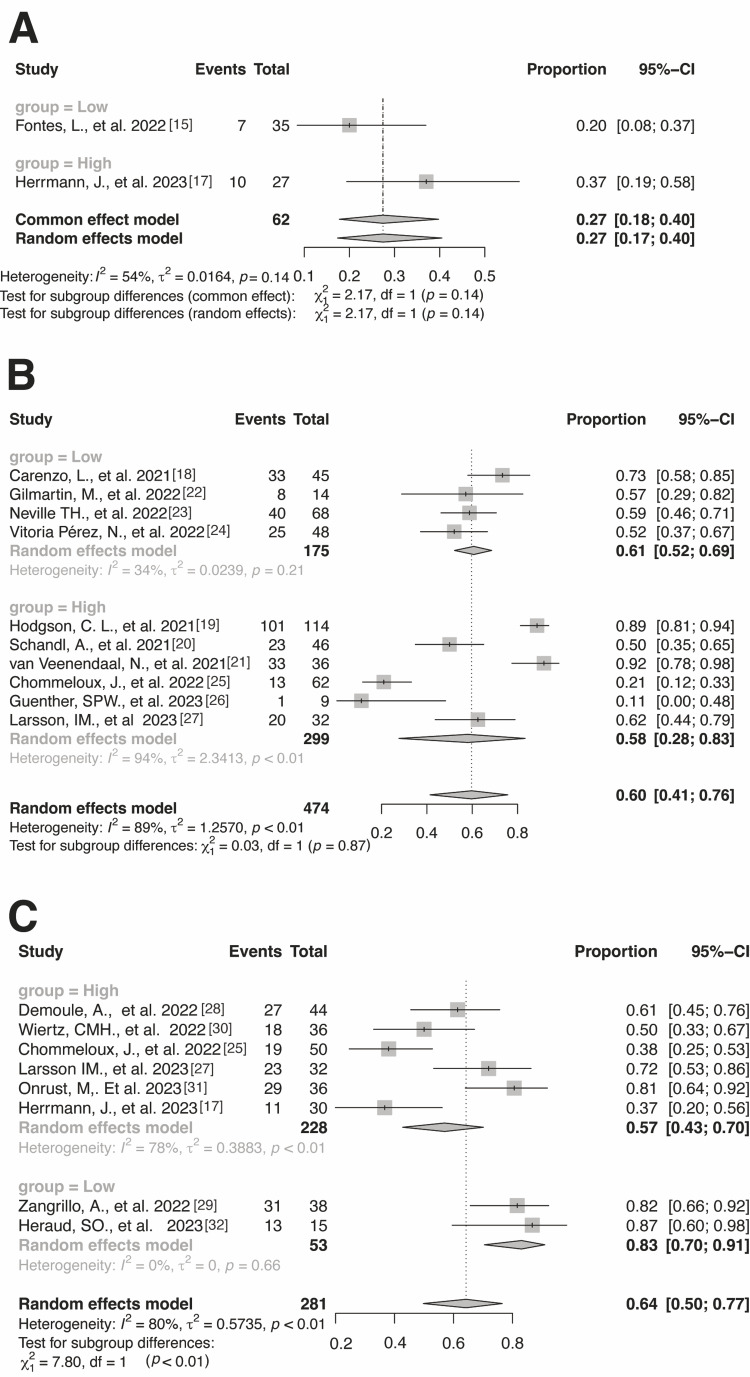
Subgroup analysis of return-to-work rates after 0–3 months (A), 4–6 months (B), and 7–12 months (C) classified according to countries with high and low integrated support

**Figure 4:**
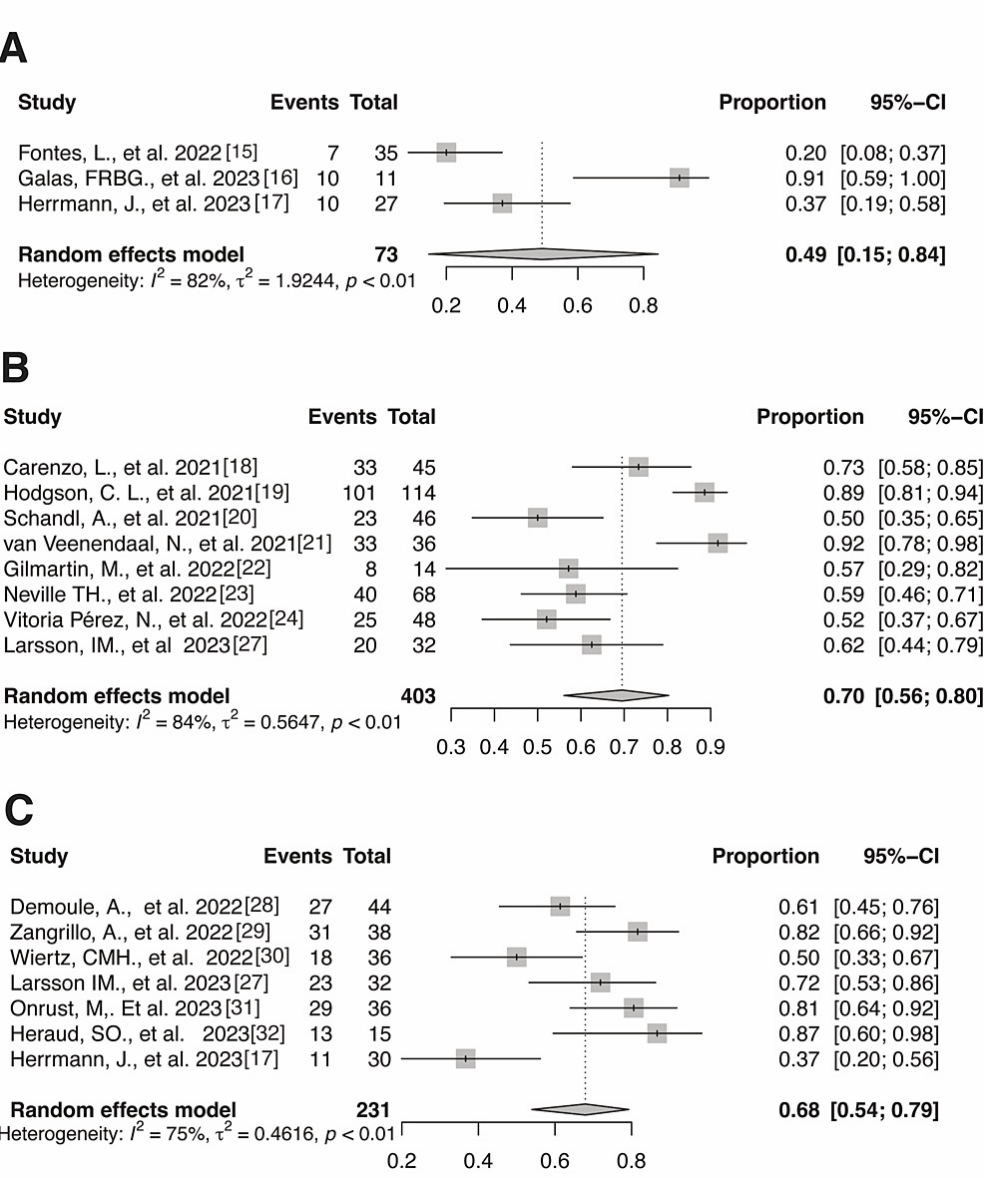
Sensitivity analysis of return-to-work rates after 0–3 months (A), 4–6 months (B), and 7–12 months (C)

We conducted a post-hoc subgroup analysis to explain the inconsistency in our findings for return-to-work prevalence in countries with highly integrated support policies compared to those with low integrated support policies. The results showed a trend toward lower return-to-work prevalence among patients in countries with lower integrated support policies 7-12 months after receiving intensive care (Figure 3). We performed a sensitivity analysis by removing studies that included patients who solely received ECMO (Figure 4). The results showed a similar trend to the main results except for the period of 7-12 months after receiving intensive care, wherein the points estimate increased in the sensitivity analysis.

Discussion

We identified 20 studies that evaluated the return-to-work prevalence among COVID-19 patients following intensive care. We found that the return-to-work prevalence in such patients gradually increased one year after discharge. The prevalence of returning to work at 0-3 months, 4-6 months, and 12 months after receiving intensive care was approximately 49%, 57%, and 64%, respectively. One year after discharge, approximately one-third of the patients did not return to work. However, the results of the reviewed studies were highly heterogeneous, and their overall quality was low.

A group of several symptoms that characterize the condition called Long COVID (e.g., fatigue, insomnia, and palpitations) and those of PICS may impact the return-to-work prevalence in patients with COVID-19. The prevalence of Long COVID has been reported in 30-50% of cases of COVID-19 [35]. Symptoms common to Long COVID may be physical and psychological [36]. One meta-analysis determined that after at least one month of formally recovering from COVID-19, the prevalence of fatigue was 0.32 (95% CI: 0.27-0.37) and the prevalence of cognitive impairment was 0.22 (95% CI: 0.17-0.28) [36]. In a follow-up study of symptoms at 2, 6, 12, and 24 months after hospitalization, fatigue was the most common symptom at six months (73%) and decreased to 21% and 36% at 12 and 24 months, respectively [33].

To our knowledge, there is no accurate information as to which of these symptoms are associated with return-to-work. Several reasons were considered with regard to employment status in the patients with COVID-19. Firstly, cognitive impairment might play a significant role in employment status; 27% of patients with severe COVID-19 had cognitive impairment six months after hospital discharge [37]. One study involving patients without COVID-19 suggested that cognitive impairment was associated with employment status 12 months after discharge [3]. Second, fatigue also may have a significant role in employment status. Third, musculoskeletal issues may be associated with employment status. A previous study has reported that patients who required mechanical ventilation were more likely to have musculoskeletal problems (38.7% vs. 2.97%) than non-critical patients after adjusting for age, sex, and length of hospital stay in the COVID-19 patients [38]. Finally, impaired pulmonary function has been associated with employment status. A past study indicated that impaired lung function was persistent at a year from discharge in patients with COVID-19 [39]. Further research is needed to determine which factors contribute the most in terms of return to work.

The return-to-work prevalence was higher in COVID-19 patients admitted to the ICU than in those without COVID-19, although our study showed a high degree of heterogeneity. A previous systematic review of return-to-work prevalence in non-COVID-19 ICU patients reported 56% (95% CI: 50-62%) [40] and 60% (95% CI, 50-59%) [4] return-to-work prevalence at the 12-month follow-up [41]. The prevalence figures reported in that study are slightly lower compared to our findings. We anticipated that it would be more challenging to return to work during the COVID-19 pandemic than under ordinary circumstances due to Long-COVID, discrimination and prejudice against those infected, and reduced economic activities due to the lockdown. It is difficult to clearly explain why the return-to-work prevalence in patients with COVID-19 was comparable to or slightly higher than that of patients without COVID-19. Changes in workstyle, such as teleworking during the pandemic, may be associated with return-to-work prevalence. Therefore, our findings may have been affected by changes in work style.

The results of this study showed a high degree of heterogeneity. This could not be explained by subgroup analysis using the level of integration support. As a result, it is difficult to provide explanations for this heterogeneity. One possible explanation is that we used the OECD report [14] published in 2010 to perform subgroup analysis classified based on the level of integrated support. However, numerous countries implemented a variety of measures during the COVID-19 pandemic, rendering the OECD classification inaccurate.

Strengths and Limitations

This is the first study to employ a meta-analysis to analyze the return-to-work prevalence in patients with COVID-19 admitted to the ICU.

Our study has several limitations. Primarily, data regarding the patients’ detailed occupational information was unavailable and the relationship between job title and return-to-work was unknown. Additionally, the certainty of the point estimates was low because of the insufficient sample size. It should be emphasized that reinstatement does not indicate that the employee is completely capable of performing the tasks when compared to their pre-COVID condition. Further research is needed to get a more accurate picture of the employment status and return-to-work prevalence in this patient population.

Clinical and Research Implications

Although severely ill patients with COVID-19 tend to return to work more quickly than those without COVID-19, approximately one-third of the patients do not return to work one year after ICU discharge, indicating that these patients need more support in terms of social integration. Workplace support is critical and may require multifaceted interventions, including health-related support, service coordination (including return-to-work programs and case management), and modification of working conditions (including changes in working hours and duties) [42]. 

A large cohort study evaluating employment status in various countries after ICU discharge is warranted. A detailed description of job characteristics, such as employment status and type, and educational level, can help us discern the actual problems and their gravity that COVID-19 patients who receive intensive care face.

## Conclusions

Based on our findings, approximately one-third of COVID-19 patients who receive intensive care do not return to work 12 months after undergoing such care; however, the overall quality of the studies we analyzed was low. These findings align with those among critically ill patients without COVID-19 infection. Long-term follow-up and integrated support are needed for these patients. Further detailed and large cohort studies that analyze the occupational type, employment status, and education levels of the patients are needed to gain deeper insights into this topic.
